# Selective oestrogen receptor antagonists inhibit oesophageal cancer cell proliferation in vitro

**DOI:** 10.1186/s12885-018-4030-5

**Published:** 2018-02-01

**Authors:** Waleed Al-Khyatt, Cristina Tufarelli, Raheela Khan, Syed Yousef Iftikhar

**Affiliations:** 1 Department of Upper GI Surgery, Royal Derby Hospital, Derby Teaching Hospitals NHS Foundation Trust, Uttoxeter Road, Derby, DE22 3NE UK; 2 Division of Medical Sciences and Graduate Entry Medicine, Royal Derby Hospital, Uttoxeter Road, Derby, DE22 3DT UK

**Keywords:** Oestrogen, Receptors, Alpha, Beta, Oesophageal, Cancer, Adenocarcinoma, Squamous, Male, Female, Sex, Hormones, Treatment

## Abstract

**Background:**

Oestrogen receptors (ER) have a well-established role to the initiation, progression and regulation of responses to treatment of breast, prostate, and lung cancers. Previous data indicates altered ER expression in oesophageal cancers (OC). However the role of ER subtypes and ER specific inhibitors in the regulation of OC progression remains unclear. This study sought to assess levels of ERα and ERβ in OC. The effects of highly selective ER antagonists on cell proliferation and apoptosis in two OC adenocarcinoma cell lines was also studied.

**Methods:**

ERα and ERβ expression profiling in paired normal oesophageal mucosa and tumour tissues (*n* = 34; adenocarcinoma *n* = 28; squamous cell carcinoma *n* = 6) was performed using quantitative reverse transcription polymerase chain reaction (qRT-PCR). Correlation between levels of ER with the clinico-pathological features for OC was determined. The effect of selective ER antagonists on proliferation of OE33 and OE19 OC cell lines was studied.

**Results:**

ERα and ERβ mRNA expression was significantly higher (*p* < 0.05) in tumour tissues relative to their paired normal mucosa and correlated inversely with survival outcome (*p* < 0.05). Upregulation of ERα mRNA correlated with higher pathological T-stage (*p* < 0.05) and lymph node metastasis (*p* < 0.05) while ERβ mRNA upregulation correlated with positive vascular invasion (*p* < 0.05). A significant concentration-dependent inhibition of proliferation in OE33 and OE19 cell lines was induced by a highly-selective ERα antagonist (MPP) and an ERβ specific antagonist (PHTPP) (*p* < 0.05). Moreover, anti-oestrogens induced cell death through stimulation of apoptotic caspase activity.

**Conclusion:**

These findings indicate that the ER system is involved in OC progression and thus may provide a novel target for the treatment of OC.

**Electronic supplementary material:**

The online version of this article (10.1186/s12885-018-4030-5) contains supplementary material, which is available to authorized users.

## Background

Oesophageal cancer (OC) is the eighth most common cancer and the sixth most common cause of cancer mortality worldwide [1]. Despite developments in treatment modalities, estimated overall five-year survival rate for patients with OC is still poor [2, 3]. It is evident that surgery alone is not a curative option for all stages of OC and additional adjunctive treatment modalities are needed [4, 5].

One of the characteristic features of OC, especially oesophageal adenocarcinoma (AC) is a persistence gender bias over several decades, in all races and across the world [6]. It occurs more frequently in males than in females, with a male to female ratio of 5–10:1, a fact that remains unexplained [7–9]. Besides, most published evidence fails so far to address any significant difference in exposure to known risk factors for the disease [10]. Instead, it is suggested that the hormonal milieu may play a possible role in this gender bias [11–15]. In support of this, the Women’s Health Initiative (WHI) study identified that the risk of developing OC is lower in pre- and peri-menopausal women compared to postmenopausal women while early menopause is associated with an increased risk of developing oesophageal AC [16]. Women who undergo intended curative resection of OC tend to have better overall survival compared with men [17]. These cumulative observations have led us to hypothesize that oestrogen signalling pathways play a role in the biological behaviour of OC.

In addition to the its roles in a diverse range of body tissues, oestrogens e.g. 17-β oestradiol are implicated in the development and progression of cancers, most obviously in breast cancer [18]. Recent reports also demonstrate involvement of oestrogen signalling in the carcinogenesis of non-classical oestrogen-sensitive tissues including colon, prostate, lung, skin, and brain [19–23]. The complex biological effects of oestrogens are mediated by two distinct receptor subtypes - ERα and ERβ (ER) and involve crosstalk between many proteins and signalling pathways [24, 25]. ER expression profiles in cancers of the breast, colon, skin, prostate and lung have been investigated extensively [26–30] and a probable role for ER in OC is suggested in a few studies on the basis of protein expression [31, 32]. While functional involvement of ER in OC is not well understood, the selective oestrogen modulator (SERM) tamoxifen appears to have an antiproliferative effect and to enhance cytotoxicity of conventional chemotherapy [32–34]. Thus there is a need to further probe mechanisms by which ER contribute to OC progression. This study addresses the notion that ER play a role in the biological behaviour of OC providing evidence for their potential utility as therapeutic targets in this malignancy OC.

## Methods

### Patient cohort

Joint ethical approval for the research protocol (08/H040/50) was acquired from the Derbyshire Research Ethics Committee and Derbyshire Hospitals Research and Development office. Written, informed consent was obtained from all patients included in this study. OC samples and matched normal tissue taken from adjacent macroscopic mucosa from the same patient were collected from resected OC specimens of 34 patients (adenocarcinoma - *n* = 28; squamous cell carcinoma - *n* = 6) who underwent oesophagectomy between January 2011 and January 2013. Normal samples were microscopically examined by a consultant pathologist to confirm normal features.

### Cell lines

Two human oesophageal cell lines (OE19 - a male adenocarcinoma and OE33 - a female adenocarcinoma, Sigma-Aldrich, Poole, UK) were used in this study. Cells were routinely cultured at 37 °C with 5% CO_2_ in the presence of penicillin (10,000 U/ml), and streptomycin (100 μg/ml) using RPMI-1640 media supplemented with 10% fetal calf serum (FCS). The presence of ERα and ERβ receptors in OE19 and OE33 cell lines was confirmed by immunofluorescence staining using an anti-ERα antibody (Santa Cruz, CA, USA) and anti-ERβ antibody (Novacastra, Newcastle, UK).

### mRNA analysis by qRT-PCR

Total RNA was extracted from tissue samples (30 mg), ground in liquid N_2_ with a pestle and mortar and from cell lines (10^4^ cells) using the RNeasy Mini kit method (QIAGEN, UK) as per manufacturer’s protocol. 300 ng of total RNA was reverse transcribed with (+RT) or without (−RT) reverse transcriptase (RT) using the high-capacity cDNA reverse transcription kit (Life Technologies, Paisley, UK). 2 μl of cDNA were amplified by real time PCR with commercially available TaqMan assays (Life Technologies, Paisley, UK) for *ESR1* (Hs00174860_m1), *ESR2* (Hs01100353_m1), and the reference genes *GAPDH* (Hs02758991_g1), *PGK1* (Hs00943178_g1), and *ACTB* (Hs01060665_g1) in a Chromo 4 thermal cycler (Bio-Rad Laboratories LTD, Hemel Hempstead, UK). Expression of *ESR1* and *ESR2* was quantified relative to the geometric mean of three reference genes and reported as relative to max using the GenEX software Version 5 (MultiD, DE) in accordance with MIQE guidelines [35] (Additional file 1: Figure S1).

### Immunohistochemistry

Immunohistochemistry (IHC) slides were prepared in the Histopathology Department at the Royal Derby Hospital. Normal mucosa and OC samples were stained using ERα and ERβ antibodies (NCL-L-ER-6F11 and 6007907, respectively, Novacastra, Newcastle, UK). ERα and ERβ positive breast cancer samples were used as positive controls. The ‘H-score method was used to measure the strength of ER-staining in normal oesophageal mucosa) and matched tumour samples [36]. Positive staining was defined as an H-score ≥ 10 in this study.

### Proliferation and cell death assays

In preparation for cell proliferation assays, cells were cultured at a final cell number of 50,000 cells/ ml in phenol red-free RPMI media (Sigma-Aldrich, Poole, UK) to eliminate the weak oestrogenic effect of this indicator. This media was supplemented with 10% stripped FCS to remove any steroids in the serum. Cells were cultured in the absence or presence of 17β-estradiol (E2), an ERα and ERβ agonist; the highly selective ERα antagonist *1,3-Bis(4-hydroxyphenyl)-4-methyl-5-[4-(2-piperidinylethoxy)phenol]-1H–pyrazole dihydrochloride* (MPP), or ERβ antagonist *4-[2-Phenyl-5,7-bis (trifluoromethyl) pyrazolo[1,5-a]pyrimidin-3-yl]phenol* (PHTPP) (Tocris Bioscience, Bristol, UK). The 5′-bromo-2′-deoxyuridine (BrdU) cell proliferation assay kit (Roche-Applied-Science, Burgess Hill, UK) was used to measure replication of genomic DNA as an indirect parameter of the cell proliferation rate. The Caspase-Glo 3/7 apoptosis assay (Promega, Southampton, UK) and the lactate dehydrogenase activity (LDH) assay (Sigma-Aldrich, Poole, UK) were used to determine the cell proliferation rates in the presence of the MPP or PHTPP.

### Statistical analysis

For qRT-PCR on primary tissues, the two-tailed Wilcoxon signed rank test was used for matched cases while the two-tailed Mann-Whitney *U* test was used for non-matched variables. Either the two-tailed Mann-Whitney *U* test or Kruskal-Wallis test, as appropriate, was used to establish relationships between hormone levels, ER mRNA and clinico-pathological features. Data for proliferation assays of the two cell lines is expressed as mean ± SD of three replicates. Two-tailed Student’s t-test was used for comparison of two groups. Comparison of multiple groups was performed using analysis of variance (ANOVA) followed by Dunnett’s or Bonferroni’s post-hoc test. Statistical differences were calculated using SPSS Statistics^®^ for Windows™ v21 software from IBM SPSS Statistics (Feltham, UK) and GraphPad Prism^®^ v6 (La Jolla, CA, USA). A value of *p* ≤ 0.05 was considered as statistically significant.

## Results

### ERα and ERβ mRNAs are increased in oesophageal tumours

To study the expression of ER in OC, primary tissues were collected from 34 OC patients (Table 1). Median age was 65 years (range, 30–79 years). There were 28 males and 6 females with a male:female ratio (5.7:1). Twenty eight patients had oesophageal AC and six patients had oesophageal SCC. One-year disease-specific survival was 73.5%. Twenty-five (74%) patients had received neo-adjuvant therapy.

**Table 1:** Patients’ Characteristics

Patients recruited		34
Median age (years)		65 (range, 30–79)
One-year disease-specific survival		73.5%
Gender	Male	28 (83%)
	Female	6 (17%)
Histology	Adenocarcinoma	28 (76%)
	Squamous cell carcinoma	6 (24%)
Tumour depth (T-stage)	T1	8 (24%)
	T2	3 (9%)
	T3	23 (67%)
Nodal involvement	No (N0)	15 (44%)
	Yes (N1)	19 (56%)
Tumour differentiation	Moderate	25 (74%)
	Poor	9 (26%)
Vascular invasion	No	21(62%)
	Yes	13 (38%)
Barrett’s Metaplasia	No	13 (46%)
	Yes	15 (54%)
Circumferential resection margin	Not involved	23 (68%)
	Involved	11 (32%)
Preoperative chemotherapy	No	9 (26%)
	Yes	25 (74%)

**Fig. 1:**
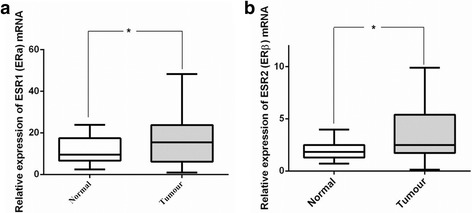
ER mRNA expression increases in oesophageal cancer. **a** Before-and-after plot demonstrates the expression of *ESR1* (ERα) mRNA in normal mucosa and oesophageal tumour samples for individual patients with oesophageal cancer (N = 34). **b** Box and whisker plot demonstrates the overall expression of *ESR1* (ERα) mRNA in normal mucosa and oesophageal tumour samples for 34 patients with oesophageal cancer. There is significant up-regulation of *ESR1* (ERα) mRNA in oesophageal tumour samples in comparison to matched normal mucosal samples (*p = 0.035, Wilcoxon matched pairs signed ranked test).**c** Before-and-after plot demonstrates the expression of *ESR2* (ERβ) mRNA in normal mucosa and oesophageal tumour samples for individual patients with oesophageal cancer (*N* = 34). **d** Box and whisker plot demonstrates the overall expression of *ESR2* (ERβ) mRNA in normal mucosa and oesophageal tumour samples from 34 patients with oesophageal cancer. There is significant up-regulation of *ESR2* (ERβ) mRNA in oesophageal tumour samples in comparison to matched normal mucosal samples (*p = 0.017, Wilcoxon matched pairs signed ranked test)

Increased expression of *ESR1* (ERα) mRNA in oesophageal tumours relative to the matched normal tissue was observed in 21/34 patients (Fig. 1a). Overall there was a significant upregulation of *ESR1* (ERα) mRNA in oesophageal tumour samples in comparison to matched normal mucosal samples (*p* = 0.035) (Fig. 1b). Similar findings were obtained for *ESR2* (ERβ) mRNA where increased expression was detected in tumours samples from 24 patients (Fig. 1c). The difference in expression between tumours and matched normal samples within the cohort was statistically significant (*p* = 0.017) (Fig. 1d).

### There is ERβ but no ERα expression at the protein level

H-scores for ERα and ERβ expression in tumour and normal mucosa samples (*N* = 34) demonstrated that only one normal mucosa sample had mild ERα staining (H-score = 10) and one tumour sample expressed mild ERα positivity (H-score = 30). The rest of the samples (*n* = 28) were negative for ERα staining in both normal mucosa and OC. On the other hand, ERβ receptor expression was detected in normal mucosa of 21 (70%) cases while only 14 (40%) tumour samples were ERβ positive but this difference was not significant (*p* = 0.29).

### ER mRNA expression has prognostic significance

**Fig. 2:**
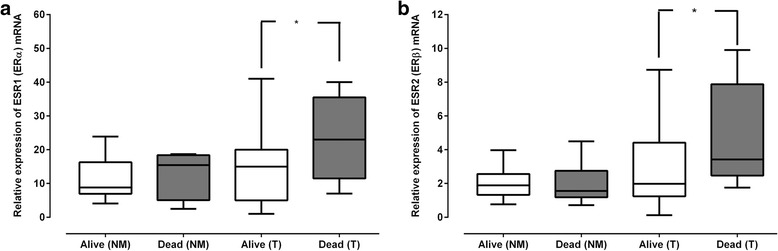
There is an inverse association between *ESR1* (ERα) and *ESR2* (ERβ) mRNA and one-year disease specific survival. **a** Box and whisker plot demonstrates the association of *ESR1* (ERα) mRNA expression in normal mucosa and oesophageal tumour samples from patients with oesophageal cancer with one-year disease specific survival, (*p = 0.046, Mann-Whitney U test). **b** Box and Whisker plot demonstrates the association of *ESR2* (ERβ) mRNA expression in normal mucosa and oesophageal tumour samples from patients with oesophageal cancer with one-year disease specific survival, (*p = 0.023, Mann-Whitney U test)

To evaluate the prognostic significance of ER mRNA expression in OC, the association of ER mRNA expression with the clinico-pathological characteristics of OC patients recruited in this study (Table 1) was analysed. When ER mRNA levels were compared to the 1-year disease-specific survival (DSS) a significant inverse association was noted, whereby upregulation of both *ESR1* (ERα; *p* = 0.046) (Fig. 2a) and *ESR2* (ERβ; *p* = 0.023) (Fig. 2b) mRNA was observed in OC samples from patients with 1-year DSS less than 12 months from their indexed date of surgery in comparison to OC samples from patients who were still alive.

The association between the expression patterns of *ESR1* (ERα) and *ESR2* (ERβ) mRNA and clinico-pathological features of OC are summarised in Table 2. There was no significant gender-based difference in the expression of *ESR1* (ERα) at OC (*p* = 0.37) and normal mucosal samples (*p* = 0.2). Similarly, there was no significant difference in the expression of *ESR2* (ERβ) mRNA in OC samples (*p* = 0.37) nor normal mucosal samples (*p* = 0.31) among male and female patients. However, there was a significant upregulation of *ESR1* (ERα) mRNA in OC samples from patients who had T3 tumours in comparison to OC samples from patients who had T1 tumours (*p* = 0.02). There was no significant difference in *ESR1* (ERα) mRNA expression in normal mucosal samples in association with tumour depth (*p* = 0.24). Furthermore, *ESR2* (ERβ) mRNA expression in T3 tumours was comparable to that of T1 tumours (*p* = 0.085). Neither was there any significant difference in the expression of *ESR2* (ERβ) mRNA in normal mucosal samples from patients who had T1 and T3 tumours (*P* = 0.53).

**Table 2:** The association between ESR1 (ERα) and ESR2 (ERβ) mRNA expression and clinico-pathological characteristics

Variable		No.	*ESR1* (ERα) mRNA expression				*ESR2* (ERβ) mRNA expression			
			Normal mucosa		Tumour		Normal mucosa		Tumour	
			Median (*IQR*)	*P* ^b^	Median (*IQR*)		Median (*IQR*)	*P* ^b^	Median (*IQR*)	*P* ^b^
Gender	Female	6	11.8 (5.7, 24.5)	0.2	14.6 (6.9, 58.1)	0.37	1.7 (1.1, 2.3)	0.31	4.1 (1.3, 5.9)	0.37
	Male	28	6.9 (7.2, 17.8)		15.4 (5.6, 23.2)		1.8 (1.3, 3.0)		2.1 (1.8, 5.0)	
Histology	AC	28	9.5 (6.5, 18.5)	0.4	14.3 (5.5, 23.6)	0.055	1.8 (1.3, 2.5)	0.39	2.1 (1.5, 5.9)	0.16
	SCC	6	11.2 (6.9, 15.3)		22.6 (18.4, 25.2)		1.7 (1.1, 2.5)		3.1 (2.1, 6.0)	
T-stage^a^	pT1	8	8.4 (7.6,14.2)	0.24	9.5 (3.3, 15.8)	**0.02**	1.7 (1.2, 3.6)	0.53	2.0 (0.9, 5.4)	0.085
	pT2	3	5.7 (2.5, 13.8)		5.7 (5.3, 8.9)		0.8 (0.7, 3.5)		1.7 (0.5, 1.8)	
	pT3	23	14.7 (6.3, 18.7)		20 (13, 31)		1.9 (1.35, 27)		3.1 (1.8, 5.6)	
LN status^a^	N0	15	7.8 (6.1, 13.4)	**0.01**	9.7 (5.4, 19.7)	**0.02**	1.4 (1.3, 2.7)	0.15	2.0 (1.0, 5.3)	0.11
	N1	19	15.9 (7.8, 21.3)		18.2 (7.3, 40.5)		1.9 (1.4, 2.4)		3.1 (1.8, 7	
Grade^a^	Moderate	25	10.2 (6.7, 18.4)		15.3 (6.0, 22.5)		1.8 (1.2, 2.7)		2.8 (1.8, 6.1)	
	Poor	9	7.8 (5.4, 16.3)		19.7 (6.0, 36.6)		1.9 (1.3, 2.6)		1.8 (1.0, 5.1)	
VI^a^	No	21	8.8 (6.2, 22.3)	0.42	14.8 (5.1, 21.4)	0.07	1.9 (1.3, 2.9)	0.41	2.0 (0.9, 4.4)	**0.01**
	Yes	13	10.2 (7.8, 16.9)		17.0 (10.7, 28.1)		1.8 (1.3, 2.3)		3.3 (2.9, 7.1)	
BM	Yes	13	13.4 (6.4, 21.3	0.26	14.6 (6.1, 18.7)	0.31	2.1 (1.3, 5.9)	0.12	1.7 (1.2, 2.9)	0.054
	No	15	6.3 (2.5, 16.6)		14.8 (5.1, 44.4)		1.9 (1.3, 2.3)		2.8 (1.7, 5.5)	
CRM	No	23	13.4 (6.7, 21.3)	0.21	14.8 (5.7, 20.3)	0.12	1.9 (1.2, 2.7)	0.44	2.0 (1.0, 7.2)	0.09
	Yes	11	8.6 (6.0, 16.6)		22.5 (7.3, 25.7)		1.8 (1.3, 2.4		2.1 (1.5, 3.1)	

*AC* oesophageal adenocarcinoma, *SCC* squamous cell carcinoma, *pT* stage is tumour’s depth, *LN* status is lymph node involvement, *BM*, Barrett’s metaplasia, *CRM*, circumferential resection margin, *IQR* interquartile range

^a^The 7th TNM Classification of Malignant Tumours proposed by the AJCC/UICC (Sobin LH, 2010)

^b^ Analysis performed Mann Whitney U test and Kruskal-Wallis test as appropriate

There was an upregulation of *ESR1* (ERα) mRNA expression in OC and normal mucosa samples from patient with nodal positive disease (N1) compared to its expression in samples from patient who had no nodal involvement (N0) (*p* = 0.01 and 0.02, respectively). In contrast, there was no significant association between the expression of ESR2 (ERβ) mRNA and nodal status either in at tumours or in normal mucosa (*p* = 0.15 and 0.11, respectively).

There was no significant association between *ESR1* (ERα) mRNA or *ESR2* (ERβ) mRNA and vascular invasion (VI) at the normal mucosal level (*p* = 0.42 and *p* = 0.41, respectively). In contrast, there was increased expression of *ESR2* (ERβ) mRNA in OC samples from patients who had VI in comparison to cancer samples from patient who had no VI (*p* = 0.01). Likewise, there was increased expression of *ESR1* (ERα) mRNA in tumours with VI compared to tumours with no VI but was not significant (*p* = 0.07). Furthermore, there was no significant association (*p* > 0.05) between ER mRNA expression and tumour differentiation, circumferential resection margin, or Barrett’s metaplasia.

### ER antagonists induce inhibition of cell proliferation in oesophageal cancer cell lines

**Fig. 3:**
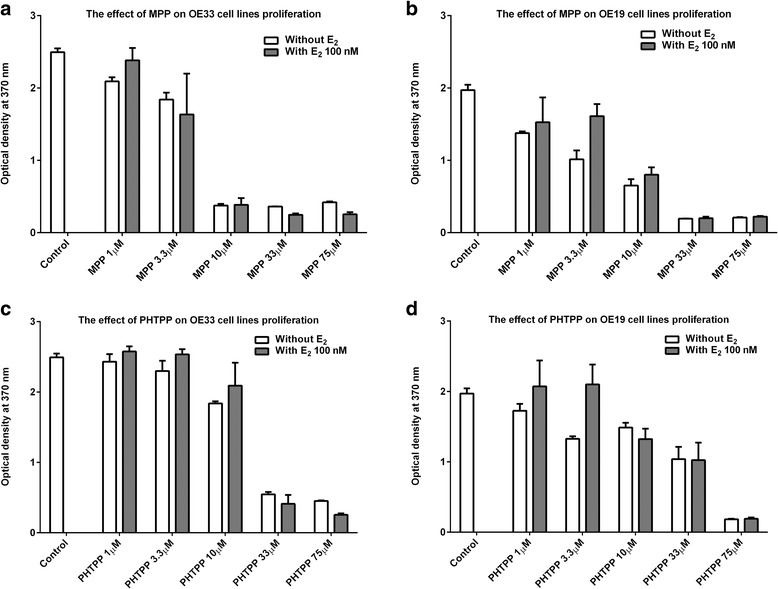
MPP and PHTPP affect proliferation of OE33 and OE19 cells. Bar chart demonstrates the effect of increasing dose of MPP and PHTPP on OE33 and OE19 cell line proliferation. Cells were seeded in triplicate using a 96-well plate at a density of 5000 cells/100 μl (without E2). At the 24 h time point, 5 different concentrations of MPP (1 μM, 3.3 μM, 10 μM, 33 μM and 75 μM) or PHTPP (1 μM, 3.3 μM, 10 μM, 33 μM and 75 μM) were added to their corresponding wells. Another set of triplicates of OE33 and OE19 cells were incubated with MPP (1 μM, 3.3 μM, 10 μM, 33 μM and 75 μM) or PHTPP (1 μM, 3.3 μM, 10 μM, 33 μM and 75 μM) grown in similar conditions, with the only exception being that E2 was added (with E2) at the 48 h time point. The proliferation rate for OE33 and OE19 cell lines was evaluated using the BrdU proliferation assay at 72 h time point. **a** MPP (without E2) showed dose-dependent inhibition of OE33 cell line proliferation (*p* < 0.0001). Adding E2 after 24 h (MPP + E2) produced no changes in the proliferation rate. **b** MPP (without E2) showed dose-dependent inhibition of OE19 cell line proliferation (*p* < 0.0001). Adding E2 after 24 h (MPP + E2) lead to increase of proliferation of OE19 cell line incubated with low concentrations of MPP (1 μM and 3.3 μM) only (*p* < 0.05). **c** PHTPP (without E2) showed dose-dependent inhibition of OE33 cell line proliferation (*p* < 0.0001). Adding E2 after 24 h (PHTPP + E2) produced no changes in the proliferation rate. **d** PHTPP (without E2) showed dose-dependent inhibition of OE19 cell lines proliferation (*p* < 0.0001). Adding E2 after 24 h (PHTPP + E2) lead to increase of proliferation of OE19 cell line incubated with low concentrations of PHTPP (1 μM, and 3.3 μM) only (*p* < 0.05)

To further investigate whether ER are potential therapeutic targets in the context of EC, in vitro experiments were performed using the oesophageal cell lines OE33 and OE19 cell lines. Firstly the effects of the ER agonist, E2, on cell proliferation were analysed. Neither stimulatory nor inhibitory effects of E2 (1, 10 and 100 nM) on OE33 and OE19 cells were observed (Additional file 1: Figure S2). In contrast, the use of antagonists specific for ERα (MPP; Fig. 3a and b) or ERβ (PHTPP; Fig. 3c and d) significantly inhibited OE33 and OE19 cell proliferation in a concentration-dependent fashion. Addition of E2 to the OE19 cell lines incubated with low concentrations of MPP (3.3 μM; Fig. 3b) or PHTPP (3.3 μM; Fig. 3d) at the 48 h time point, demonstrated slight but significant stimulation of proliferation (*p* = 0.01). However, E2 (100 nM) produced no effect on the proliferation of OE33 cells incubated with MPP (Fig. 3a) or PHTPP (Fig. 3c) (*p* > 0.05).

### ER antagonists promote apoptosis

**Fig. 4:**
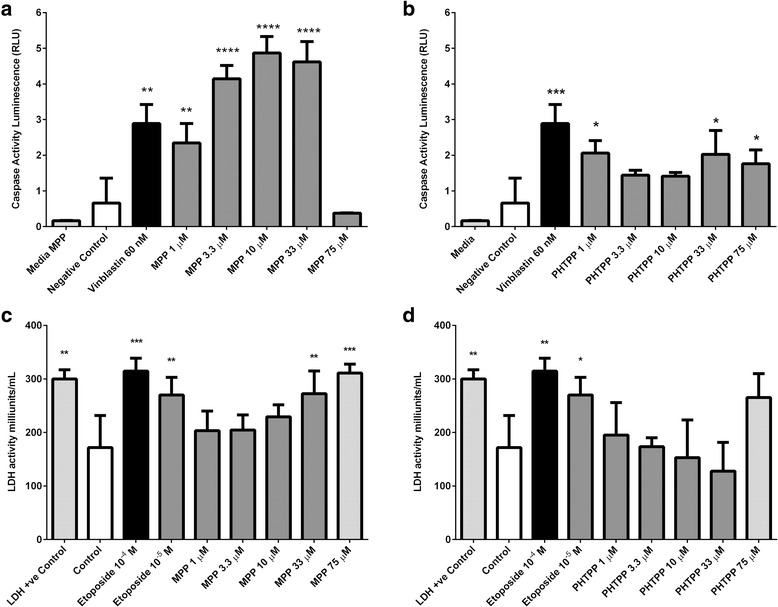
Effect of MPP and PHTPP on the caspase-3/ caspase-7 activity and on the lactate dehydrogenase activity (LDH) of OE33 cell lines. Cells were treated with the indicated concentrations of MPP and PHTPP for 48 h, and then caspase activity was determined using Caspase-Glo 3/7 assay while LDH activity was determined using LDH activity assay. Data are presented as mean ± SD of two independent experiments. **a** There was a significant increase in caspase 3/7 activity of cells treated with MPP 1 μm, 3.3 μm, 10 μm, and 33 μm compared to the negative control (****p < 0.0001). **b** There was a significant increase in caspase 3/7 activity of cell treated with PHTPP 1 μm, 33 μm, and 75 μm compared to the negative control (*p < 0.05). **c** There was a significant increase in the LDH activity of cells treated with MPP 33 μm and 75 μm compared to the negative control (***p < 0.001). **d** Only cells treated with PHTPP 75 μm showed increased LDH activity compared to the negative control, however the difference did not reach statistical significance (*p* = 0.24)

To investigate the mechanism underlying the reduction in OC cell line proliferation induced by ER antagonists, further work was performed using OE33 cells to test for Caspase3/7 and lactate dehydrogenase activities. There was significant increased activity of caspase 3/7 of OE33 cell lines treated with MPP 1 μM, 3.3 μM, 10 μM, and 33 μM (*p* < 0.0001) but not in cells incubated with MPP 75 μM (*p* = 0.5) compared to OE33 cells cultured with no added drugs (Fig. 4a). Similarly, the activity of caspase 3/7 of OE33 cell lines was significantly raised when cells were incubated with PHTPP 1 μM, 33 μM, and 75 μM compared to the negative control (*p* < 0.05) (Fig. 4b). Increased activity was also noted in cells treated with PHTPP 3.3 μM and 10 μM, however the results did not reach statistical significance (*p* = 0.12; Fig. 4b).

In the presence of MPP, there was an increase in the LDH activity in the supernatant taken from OE33 cell lines incubated with MPP 33 μM and 75 μM (*p* < 0.001) compared to the negative control (Fig. 4c). There was no change in LDH activity in supernatants isolated from cells incubated with PHTPP at 1 μM, 1 μM, 3.3 μM, 10 μM, and 75 μM (*p* = 0.9) (Fig. 4d).

## Discussion

This study describes investigations of ER expression in OC samples versus normal mucosa and potential prognostic implications. It also demonstrates the effect of highly selective ER antagonists on OC cell proliferation in vitro and the possible underlying mechanism behind reduced proliferation rates. Initially, the expression of ER was measured using qRT-PCR in normal mucosa and tumour samples from patients with potentially resectable OC. The measurement of mRNA levels demonstrated that both ER subtypes are expressed in normal mucosa and tumour samples. Additionally, there was a significant upregulation of ERα and ERβ mRNA expression in OC biopsies compared to their matched mucosal samples. It was also demonstrated that ERα and ERβ may have a potential prognostic role on the basis that mRNA levels for both receptors have a significant inverse association with one-year disease-specific survival and certain clinico-pathological features. In vitro experiments performed using oesophageal cell lines OE33 and OE19 demonstrated significant concentration-dependent inhibition of cell proliferation using selective ER antagonists in both cell lines.

ER have an essential role in the proliferation and differentiation of normal tissues and consequently oestrogen signalling may also play a role in the dysregulation of these processes in cancer cells [37]. In addition, altered expression of ER is considered as an initial step towards the development of certain cancers [24]. For instance, loss of ERβ increases proliferation of colon cancer cell lines [38] while increased ERβ expression leads to cell cycle arrest [39, 40]. In the breast, ERα mediates the proliferative effect of E2 and ERβ has anti-proliferative effects [41, 42]. In prostate cancer, the expression of ERβ undergoes gradual reduction in the expression from normal tissue to benign prostatic hyperplasia towards invasive prostate cancer [43]. Furthermore, the re-introduction of ERβ into prostatic cancer cell lines was associated with decreased proliferation and increased apoptosis [22]. A recent study from Germany investigated the significance of ERα expression in non-small cell lung cancer (NSCLC) samples from 64 patients who underwent radiotherapy treatment [44]. It was found that ERα expression in NSCLC inversely associated with disease-free and overall survival [44]. The number of studies investigating ER status in OC is scarce and the results are rather conflicting and inconclusive. Nevertheless, it was suggested that ERβ is the predominant receptor in oesophageal normal mucosa and OC while ERα is only expressed at very low levels [31, 45–49]. The presence of ER subtypes at the mRNA level in normal mucosa has prompted us to postulate that ER play a role in normal oesophageal function. Moreover, the observation of increased expression of ER subtypes in tumour samples may also indicate a biological role in OC development.

It has been suggested that oestrogens confer protective effects on the development of OC. In this study, the effect of E2 on OC proliferation in vitro demonstrated no significant changes in proliferation rates of the OE33 and OE19 cell lines when cells were incubated with increasing concentrations of E2. However, there was significant inhibition of OE33 and OE19 cell lines by increasing the concentrations of a highly selective ERα antagonist (MMP) and an ERβ antagonist (PHTPP). In addition, it was also demonstrated that the mechanism behind this reduction in cell growth rate is the initiation of a programmed cell death rather than a direct cytotoxic effect. These findings support our hypothesis that oestrogen signalling pathways may have a role in the biological behaviour of OC. However, further studies of cell-cycle analysis are necessary to distinguish the molecular mechanisms behind these findings [50, 51].

The effect of E2 via ER is influenced by several factors. Hence, the finding of no altered proliferation rate in response to E2 may be due to the fact that OC cell lines express ERα and ERβ at similar levels and activation of one receptor could have antagonised the function of the other receptor [18, 24] especially if the ER subtypes have opposing actions. On binding of E2 with ER, the end result is also affected by the type of co-regulators recruited into action. For instance, if a co-suppresser like *Repressor of oestrogen receptor Activity* (REA) is bound to the E2/ER complex, it will lead to inhibition of activation of ERE and gene transcription [24, 51, 52]. Lastly, the absence of an E2 effect can also be explained by post-translational modifications where the E2/ER complex is promptly metabolised by ubiquitination or phosphorylation [24, 53].

In this study, the reason for the lack of the expression of ERα protein is unclear and may be theoretically explained by stating that ERα (*ESR1*) gene is simply a non-functional gene. However, this explanation seems rather naïve given that all normal mucosal and tumour samples used in this study demonstrated variable levels of ERα mRNA. Moreover, there was altered expression of ERα mRNA between normal mucosa and tumour samples. In addition, both OC cell lines (OE33 and OE19) demonstrated moderate expression of ERα at the protein level. Hence, other factors might have contributed to the ERα negative status in tissue samples. For example, previous studies have suggested that monoclonal antibodies can be species and tissue-specific [54, 55]. In this study, we used mouse monoclonal antibodies for the quantification of ERα status. These antibodies were developed using a prokaryotic recombinant protein as an immunogen which corresponds to the full-length human ERα molecule. Interestingly, there was strong ERα staining in breast cancer tissue used as a positive control. Using the same antibodies, Kalayarasan et al. found no ERα expression in 45 OC specimens (SSC = 30, AC = 15) [49]. Moreover, Kawai et al. found that using monoclonal antibodies (against NH2 terminus of ERα) for quantification of ERα in NSCLC produced negative results whereas the use of polyclonal antibodies (against COOH terminus of ERα) gave positive ERα staining [28]. This may suggest that ERα isoforms localised in oesophageal tissue may lack an epitope which is specific to monoclonal antibodies [55]. This could have contributed to the lack of ERα staining in our cohort [28].

To the best of our knowledge, this study is the first to investigate the ER status in patients with OC, mainly oesophageal AC from a UK population. It also builds on other studies by Sukocheva et al. [32] and Due et al. [34] where in vitro effects using a selective ER modulator on OC cell lines confirmed anti-proliferative effects observed with Tamoxifen. However, we opted to use only MPP and PHTPP rather than Tamoxifen for an important experimental reason. The agonist/antagonist property of Tamoxifen varies among tissues [56]. For instance, Tamoxifen acts as an ER antagonist on breast tissue and inhibits breast cells proliferation, however it acts as an ER agonist (i.e., mimicking the effects of oestrogen) in bone and uterine cells [56]. Its action on oesophageal cancer cells used in Sukocheva et al. [32] and Due et al. [34] is not clearly explained whether based on its antagonist or agonist property. In comparison, MPP and PTHPP are highly selective ER antagonists and blocking them allows one to suggest that any experimental effects are likely due to the involvement of these receptors.

There are a few limitations in this study. Firstly, in vitro experiments carried out to investigate the potential role of E2 and ER do not often mimic effects in vivo. For this reason, the findings may not necessarily produce similar biological effects if experiments are run in vivo*.* Secondly, the cancer cell lines used in this study might have undergone epigenetic modifications and so this could somewhat affect the results generated [57]. Thirdly, the work conducted in this study to address the change of ER status was performed on samples obtained from patients with only potentially resectable OC. Hence, it is not known whether comparable results are still possibly obtainable if samples are collected from patients with locally advanced or metastatic disease. Finally, neither the effect of E2 or ER modulators on normal oesophageal epithelial physiology nor ER status in normal oesophageal mucosa samples obtained from patients with non-malignant oesophageal pathologies were investigated.

## Conclusion

Our findings indicate a possible role for ER in the biological behaviour of OC. We demonstrate that a significant increase of ER mRNA levels in OC which inversely correlates with survival and pathological features. Furthermore, selective blocking of ER inhibited OC cell proliferation. Further studies examining ER as novel targets for the treatment of OC are required.

## Additional file

Additional file 1: Figure S1. Identification of suitable reference genes. Reference genes expression stability was analysed using geNorm and NormFinder. **Figure S2.** E2 treatment does not alter proliferation of OE33 and OE19 cells. Bar chart demonstrates the effect of increasing dose of E2 on OE33 and OE19 cell lines proliferation. (PDF 348 kb)

## Abbreviations

+RT: Positive reverse transcribed
AC: Adenocarcinoma
E2: 17β-estradiol
ER: Oestrogen receptors
ERα: Oestrogen receptor alpha
ERβ: Oestrogen receptor beta
*ESR1*: ERα gene
*ESR2*: ERβ gene
FCS: Fetal calf serum
MPP: Highly selective ERα antagonist *1,3-Bis(4-hydroxyphenyl)-4-methyl-5-[4-(2-piperidinylethoxy)phenol]-1H–pyrazole dihydrochloride*
OC: Oesophageal cancer
OE19: Male oesophageal adenocarcinoma cell line
OE33: Female oesophageal adenocarcinoma cell line
PTHPP: ERβ antagonist *4-[2-Phenyl-5,7-bis (trifluoromethyl) pyrazolo[1,5-a]pyrimidin-3-yl]phenol*
-RT: Negative reverse transcriptase
RT-PCR: Reverse transcription polymerase chain reaction
