# Supplementary material for: Selective oestrogen receptor antagonists inhibit oesophageal cancer cell proliferation in vitro
Source: BMC Cancer. 2018 Feb 1;18:121. doi: 10.1186/s12885-018-4030-5 (PMC5796348; doi:10.1186/s12885-018-4030-5)
Supplement: Additional file 1: Figure S1. — Identification of suitable reference genes. Reference genes expression stability was analysed using geNorm and NormFinder. Figure S2. E2 treatment does not alter proliferation of OE33 and OE19 cells. Bar chart demonstrates the effect of increasing dose of E2 on OE33 and OE19 cell lines proliferation. (PDF 348 kb) [file 12885_2018_4030_MOESM1_ESM.pdf]

SUPPLEMENTARY INFORMATION

Contents

SUPPLEMENTARY INFORMATION 1

Supplementary Figure S1 2

Supplementary Figure S2 3

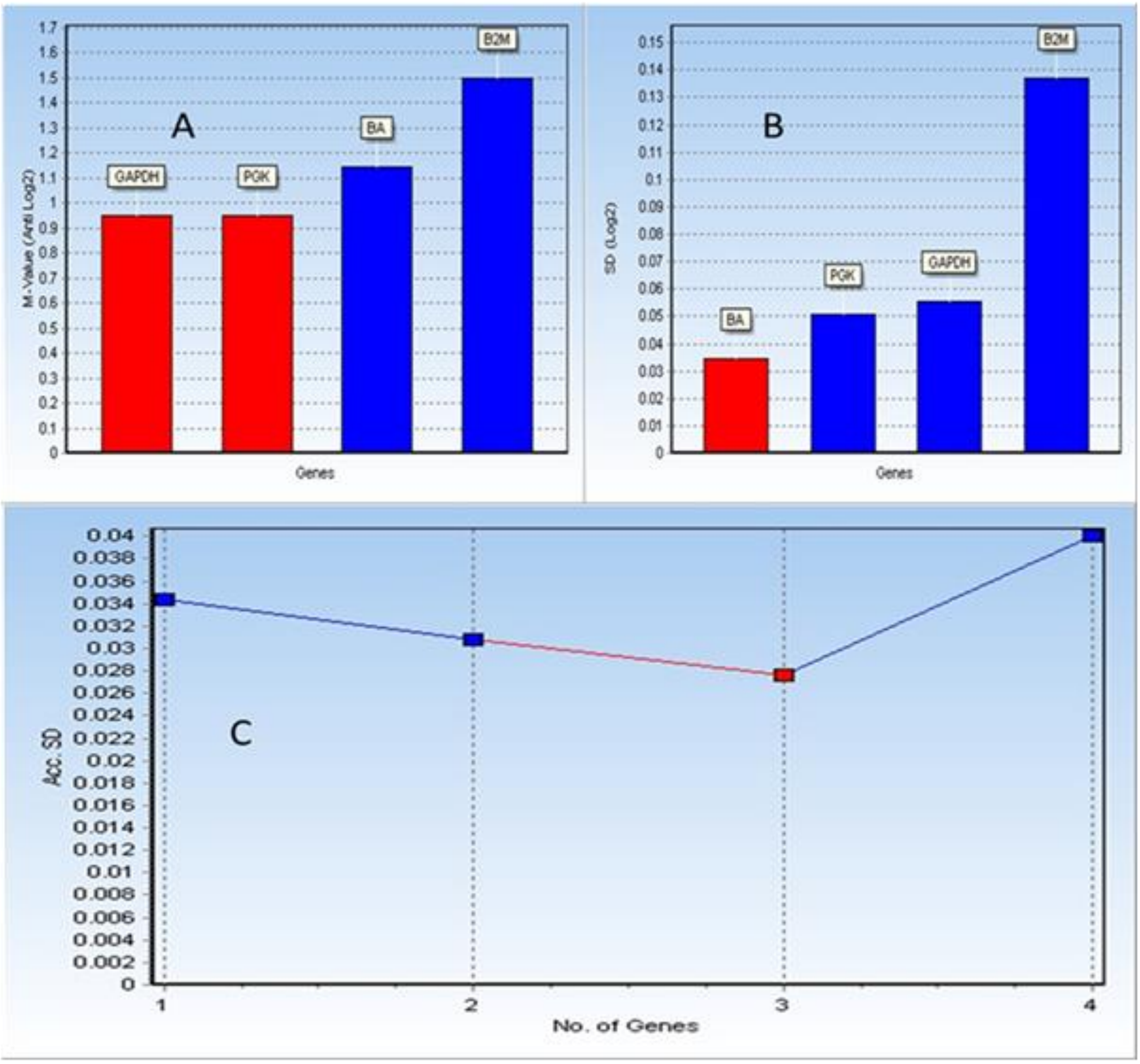

Supplementary Figure S1: Identification of suitable reference genes. Reference genes expression stability was analysed using geNorm and NormFinder. A) *PGK1* and *GAPDH* were most suitable genes for qRT-PCR normalisation, followed by BA (*ACTB*), while B2M was least stable gene using geNorm. B) BA (*ACTB*) was the most stable gene, followed by *PGK1* and *GAPDH* while *B2M* was again least stable using NormFinder. C) *PGK1, GAPDH,* and BA (*ACTB*) had the best combination accumulative standard deviation (Acc. SD = 0.028) using NormFinder.

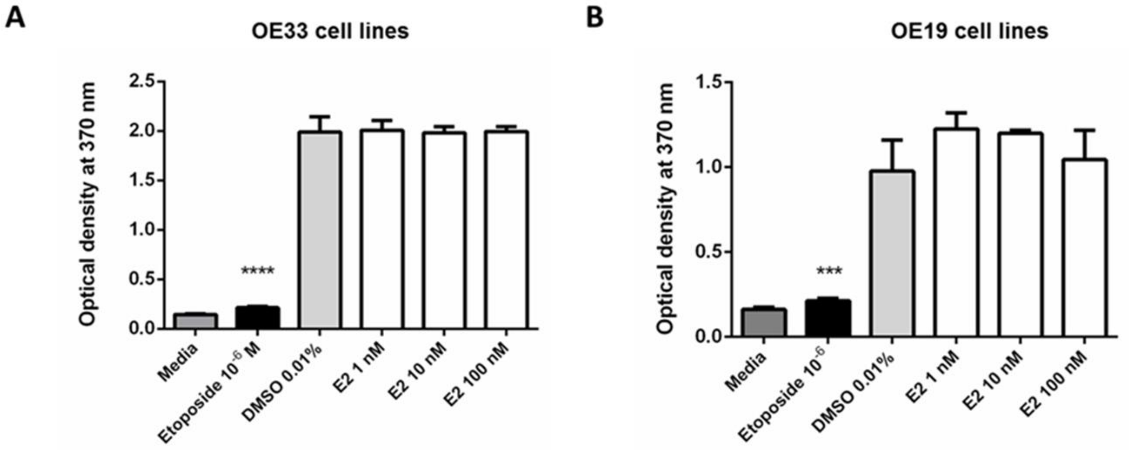

Supplementary Figure S2: E2 treatment does not alter proliferation of OE33 and OE19 cells. Bar chart demonstrates the effect of increasing dose of E2 on OE33 and OE19 cell lines proliferation. OE33 and OE19 cell lines were incubated with 3 different concentrations of E2 (1, 10 and 100 nM) at a density of 5000 cells/100 µl. The proliferation of cell lines was evaluated using 5' bromo 2' deoxyuridine proliferation assay at 72 hours' time point. Three different concentrations of E2 showed no changes on the proliferation rate for OE33 and OE19 cell lines (*p* = non-significant).
